# Seasonality affects dietary diversity of school-age children in northern Ghana

**DOI:** 10.1371/journal.pone.0183206

**Published:** 2017-08-14

**Authors:** Abdul-Razak Abizari, Fusta Azupogo, Miwako Nagasu, Noortje Creemers, Inge D. Brouwer

**Affiliations:** 1 Department of Community Nutrition, School of Allied Health Sciences, University for Development Studies, Tamale, Ghana; 2 Department of Family and Consumer Sciences, Faculty of Agriculture, University for Development Studies, Tamale, Ghana; 3 Division of Human Nutrition, Wageningen University, The Netherlands; University Sains Malaysia, MALAYSIA

## Abstract

**Background and objectives:**

Dietary diversity score (DDS) is relatively easy to measure and is shown to be a very useful indicator of the probability of adequate micronutrient intake. Dietary diversity, however, is usually assessed during a single period and little is known about the effect of seasonality on it. This study investigates whether dietary diversity is influenced by seasonality.

**Methods:**

Two cross-sectional surveys were conducted in two different seasons—dry season (October 2010) and rainy season (May 2011) among the same school-age children (SAC) in two rural schools in northern Ghana. The study population consisted of 228 school-age children. A qualitative 24-hour dietary recall was conducted in both seasons. Based on 13 food groups, a score of 1 was given if a child consumed a food item belonging to a particular food group, else 0. Individual scores were aggregated into DDS for each child. Differences in mean DDS between seasons were compared using linear mixed model analysis.

**Results:**

The dietary pattern of the SAC was commonly plant foods with poor consumption of animal source foods. The mean DDS was significantly higher (*P* < 0.001) in the rainy season (6.95 ± 0.55) compared to the dry season (6.44 ± 0.55) after adjusting for potential confounders such as age, sex, occupation (household head and mother) and education of household head. The difference in mean DDS between dry and rainy seasons was mainly due to the difference in the consumption of Vitamin A-rich fruits and vegetables between the seasons. While vitamin A-rich fruits (64.0% vs. 0.9%; *P* < 0.0001) and vitamin A rich dark green leafy vegetables (52.6% vs. 23.3%, *P* < .0001) were consumed more during the rainy season than the dry season, more children consumed vitamin A-rich deep yellow, orange and red vegetables during the dry season than during the rainy season (73.7% vs. 36.4%, *P* <0.001).

**Conclusion:**

Seasonality has an effect on DDS and may affect the quality of dietary intake of SAC; in such a context, it would be useful to measure DDS in different seasons. Since DDS is a proxy indicator of micronutrient intake, the difference in DDS may reflect in seasonal differences in dietary adequacy and further studies are needed to establish this.

## Introduction

Dietary diversity assessed as the number of food groups consumed over a reference period has been proposed as a potential proxy indicator for diet quality [1–3]. There is substantive evidence from developed countries showing that dietary diversity is indeed strongly associated with nutrient adequacy and is thus an essential element of diet quality [4–7]. Studies recently emerging from developing countries also support the association between dietary diversity and nutrient adequacy [8–12]. Studies from resource poor settings have also established an association between dietary diversity and nutritional status of children [13–16] as well as women [17–19].

Dietary diversity is usually assessed during a single time period but may be influenced by seasonality, especially in resource poor settings. Seasonality is known to influence the consumption of food [20–22]. Ferguson et al [23] reported seasonal variations in the consumption of fruits, legumes, roots, plantains and some flour among Ghanaian and Malawian preschool children. Hudelson et al [24] also observed that adequate consumption of vitamin A rich foods in Ghana is hindered by seasonality as green leafy vegetables are abundant only in the rainy season. A study from Burkina Faso confirmed that seasonality has an influence on dietary diversity score (DDS) as well as the nutritional status of women [18].

With seasonal fluctuation in agricultural production, populations relying heavily on agriculture outputs with little access to animal source foods are more likely to experience seasonal food shortages. Seasonal variations in nutrient intake have also been reported. A study in Kenya found significant improvement in intakes of calcium, zinc, iron and folate during the rainy season compared to the dry season among preschool children [25]. Whilst some studies have documented significantly higher intakes of micronutrients during the post-harvest season compared to the lean season in Burkina Faso [26,27], Savy et al [18] reported a higher DDS with a decreased nutrient adequacy in the lean season compared to the post-harvest season in rural Burkina Faso. This suggests there may be a lack of consistency in the pattern of seasonal fluctuations of dietary diversity and nutrient adequacy and more studies are needed to elucidate the effect of seasonality on DDS.

To the best of our knowledge, our present study is the first to examine the influence of seasonality on the dietary diversity of school-age children (SAC). We hypothesized that the dietary diversity score of SAC in Northern Ghana will vary between the dry season (October) and the rainy season (May). The information from the present study will be useful in informing the selection and design of context-specific strategies to reduce micronutrient deficiencies.

## Materials and methods

### Study area

The study was carried out in 2 primary schools in 2 rural communities in the Tolon district of northern Ghana between October 2010 and May 2011. The 2 communities have similar socio-demographic characteristics. The climate of the district is relatively dry, with a single rainy season that begins in April and ends by October. The dry season starts in October and ends in March with maximum temperatures occurring towards the end of the dry season [28]. The vegetation is guinea savannah characterized by tall grasses interspersed with drought resistant trees such as the shea (*Butyrospermum Parkii*) and dawadawa (*Parkia Biglobosa*) [28]. Subsistence agriculture is the main stay of the people in the district and major crops cultivated include cereals (maize, sorghum, millet and rice), legumes (soybeans, groundnuts and cowpeas) and starchy roots and tubers (cassava, yam and potato) [28].

### Study design and study population

This study was carried out within the framework of the TELFUN-Ghana dietary intervention trial [29]. A sample size of 241 was determined for the trial based on another primary outcome (iron deficiency anaemia). In brief, two communities (with primary schools) which qualified to benefit from the Ghana School Feeding Programme (SFP) but were not yet enrolled, were selected as control schools based on their similarity with the SFP pilot schools with respect to the following characteristics: number of children enrolled in school; school infrastructure; size of community; absence of market infrastructure; water and sanitation facilities and proximity to each other [29,30]. Subsequently, children who met the inclusion criteria were randomly assigned to the treatment and control arms of the dietary trial; details of the selection procedure have been published elsewhere [29,30].

We assessed the dietary diversity score (DDS) of the SAC at two different time points using a qualitative 24-hour dietary recall (24hR). The baseline assessment (October 2010) was conducted before the intervention and corresponded with the onset of the dry season and the harvest of most crops especially cereals, legumes like cowpea and some root tubers particularly sweet potatoes. On the contrary, the follow-up assessment (May 2011) was conducted at the end of the intervention and was in the rainy season coinciding with the lean period when many households have their staple food (cereal and root tubers) stocks depleted.

At baseline, anthropometric measurements (weight and height) of the children were taken and a pretested semi-structured questionnaire was used to assess the socio-demographic and economic characteristics of the children and their households. Some children were lost to follow-up (n = 13) and only children with data at both time points (n = 228) were included in the final analysis. Ethical approval for the study was obtained from the Institutional Review Board of Noguchi Memorial Institute for Medical Research, University of Ghana and Medical Research Ethics Committee of Wageningen University, The Netherlands. We obtained permission from the district administration, chiefs and opinion leaders of the respective communities. Thumb-printed informed consent was also obtained from each parent or caregiver.

### Dietary assessment

A qualitative 24hR was used to assess the dietary intake of the SAC at both time points (October 2010 and May 2011). Although the SAC were the primary subjects, mothers and caregivers were respondents for the children as children are likely to give inaccurate descriptions of their dietary intake [31,32]. Mothers and caregivers were first asked to mention all foods including drinks and snacks that were consumed the previous 24 hours (from wake-up to wake-up) preceding the survey by the index child from home and outside of the home. She was next probed for likely forgotten foods and then asked to give a detailed description of foods and beverages consumed, including ingredients for mixed dishes. To ensure intake outside the home was captured, children were asked to assist their mothers/caregivers in the recall.

### Dietary diversity score (DDS)

DDS was calculated for each child based on a set of 13 food groups namely: (1) starchy staples, (2) legumes and nuts, (3) dairy products, (4) organ meat, (5) eggs, (6) small whole fish, (7) flesh foods, (8) vitamin A rich dark green leafy vegetables, (9) vitamin A-rich deep yellow, orange and red vegetables, (10) vitamin A-rich fruits (vitamin A ≥ 120RE/100g), (11) vitamin C-rich vegetables (vitamin C ≥ 9 mg/100g), (12) vitamin C-rich fruits (vitamin C ≥ 9 mg/100g) and (13) all other fruits and vegetables. The choice of the food groups was based on Arimond et al [33]. Definition of Vitamin A rich and vitamin C-rich fruits or vegetables was based on the FAO definition [34]; subsequently based on the nutrient content of the vegetables or fruits in the Ghana/Mali Food Composition Tables [35,36], they were classified accordingly as vitamin A rich dark green leafy vegetables, vitamin A-rich fruits and vitamin A rich-deep yellow orange and red vegetables. The food items in each food group are presented in Table 1.

**Table 1:** Food groups used in the dietary diversity score with food items included in each food group.

Food group	Individual food items
All starchy staples	Guinea corn, maize, rice, wheat, sorghum, millet, plantain, yam, cassava, white sweet potato, or any other grains or foods made from these (e.g. bread, noodles, porridge or other grain products)
All legumes and nuts	Beans, peas, lentils, nuts, seeds, groundnuts, cowpea, soybean, bambara beans, pigeon peas, cashew nut, Bungu (sesami), neri (melon seeds) or foods made from these
Dairy products	Milk, powdered milk, cheese, yogurt or other milk products
Organ meat	Liver, kidney, heart or other organ meats or blood-based foods
Eggs	Chicken, duck, guinea fowl or any other egg
Small fish eaten whole	Fresh or dried fish or shellfish, anchovies
All other flesh foods	Beef, lamb, goat, rabbit, wild game, chicken, duck, guinea fowl or other birds
Vitamin A rich dark green leafy vegetables	Dark green/leafy vegetables, including wild ones + locally available vitamin-A rich leaves such as kenaf/roselle, amaranth, cassava leaves, cowpea leaves, onion leaf, jute mallow
Vitamin A-rich deep yellow, orange and red vegetables	Tomato, carrots, or sweet potatoes that are orange inside and other locally available vitamin-A rich vegetables (e.g. red sweet pepper)
Vitamin A- rich fruits VA rich ≧120RE/100g as eaten	Ripe mangoes, ripe papaya, sheanut fruits, watermelon, dawadawa pulp and other locally available vitamin A-rich fruits
Vitamin C rich vegetables (Vitamin C ≥9 mg/100g as eaten)	Baobab leaf, lettuce, green pepper, red pepper
Vitamin C-rich fruits (Vitamin C ≥9 mg/100g as eaten	Orange, banana, lemon, watermelon, guava
All other fruits and vegetables	Other fruits and vegetables, including wild fruits and vegetables, okro, onion, pineapple, apple, garden eggs, ebony fruits, blackberry, cashew fruits

At baseline and follow-up, a score of 1 was assigned if a child consumed a food item belonging to a particular food group, else 0. Individual food group scores were aggregated into DDS for each child at baseline and follow-up. Any food consumed on multiple occasions during the 24 hour period was counted only once resulting in a maximum attainable score of 13. The scoring did not consider a minimum intake for the food groups.

We also created a score for vitamin A rich fruits and/or vegetables consumption (sum score for: vitamin A-rich dark green leafy vegetables, vitamin A-rich deep yellow, orange and red vegetables and vitamin A-rich fruits), vitamin C-rich fruits/vegetables consumption (sum score for: vitamin C-rich vegetables and vitamin C-rich fruits) and animal source foods (ASF) consumption (sum score for: all dairy products, organ meat, small fish eaten whole, eggs, and all other flesh foods). The score for vitamin A-rich fruits and/or vegetable intake, vitamin C-rich fruits and/or vegetable intake and ASF intake ranged from 0–3, 0–2 and 0–5 respectively.

### Covariates

The weight and height of the children were measured following standard procedures [37]. Weight was measured to the nearest 0.1kg with a calibrated electronic scale (UNIscale; Seca) whilst standing height was measured to the nearest 0.1 cm with a microtoise (Bodymeter 208; Seca). The measurements were duplicated and averaged to reduce random instrumental error. We used the anthropometry to compute the Body Mass Index-for-age z-score (BAZ), weight-for age z-score (WAZ) and height-for-age Z-score (HAZ) of each child. The completed age of each child in years was determined using any reliable source (birth certificate, child health record, health insurance card) and the date of collection of anthropometric data. Where there was no reliable source of birth date, the parents/caregiver were asked to estimate age based on another child’s records or event on the traditional calendar (12 children) [29].

We assessed the demographic and socioeconomic characteristics of the children’s households with a pretested semi-structured questionnaire. Demographic and socioeconomic related covariates included child’s compound size (number of people living in child’s house), educational status of household head and mother as well as the occupation of household head and mother.

### Statistical analysis

Population characteristics were presented as means (standard deviations) for continuous variables and frequency (percentages) for categorical variables. Univariate analysis of variance with the MIXED procedure [38] was used to analyze the association between DDS and seasonality. Potential confounders included as covariates were a priori selected based on literature and included age, sex, nutritional status of the child (underweight), total compound size, educational status of household head and mother as well as the occupation of the household head and mother [17–19,39]. Spearman and Pearson correlation coefficient showed no multicollinearity between the confounding variables. The model was checked for effect modification by sex and age but none was statistically significant and were thus excluded in the final analysis. Besides the crude model, we created 2 multivariate models. The first multivariate model was adjusted for demographic factors (age and sex). The full model was further adjusted for socioeconomic factors of child’s household (educational status of household head, the occupation of household head and occupation of the mother). Whilst occupation of household head was modelled as farmer or other (trader and civil servant), maternal occupation was modelled as farmer, trader and other (housewife and civil servant) based on the data available. Although a priori chosen, we excluded nutritional status defined by BAZ and total compound size from the models as they had no influence on the results. Furthermore, educational status of mother and household head had low variability; we, however, modelled the educational status (literate or non-literate) of the household head which had more variability than that of the mother.

We also compared the DDS at baseline and end-line between the treatment and control arms of the feeding trial using One-Way ANOVA but found no differences between treatment and control arms of the feeding trial; hence we did not adjust for the intervention effect. The X^2^ statistic was used to determine the association between the percentage of children consuming a food group and seasonality as well as the association between DDS category (low and high) and seasonality. Although DDS is commonly categorized into tertiles (low, moderate and high), the median split (low and high) was preferred because there was little variation (DDS difference = 1) between the 33^rd^ percentile (DDS = 6) and the 67^th^ percentile (DDS = 7); even when using the tertile categorization similar to the FAO (DDS ≤ 3, DDS of 4–5 and DDS ≥6) [34], only 2 DDS categories similar to the median split were created. All statistical analyses were done using SAS 9.3 (SAS Institute Inc., Cary NC.) and a two-tailed *P*-value ≤ 0.05 at 95% confidence interval was considered statistically significant.

## Results

### Characteristics of the sample

Table 2 shows the characteristics of the study population at baseline. The mean age of the SAC was 8.1 ± 2.1 years and the mean BAZ was -0.5 ± 0.8. About 62.7% of the children were boys and the average compound size was 15.5 ± 8.7. The household heads (91.1%), as well as the mothers (69.3%), were predominantly farmers. The majority of the household heads (84.1%) and the mothers/caregivers (97.8%) of the children had never been to school.

**Table 2:** Population characteristics of the school-age children and their households.

Variable	n = 228
Age in years (mean ± SD)	8.1 ± 2.1
Total compound size (mean ± SD)	15.5 ± 8.7
BAZ^1^ (mean ± SD)	-0.5 ± 0.8
HAZ^2^ (mean ± SD)	-1.4 ± 1.2
WAZ^3^ (mean ± SD)	-1.2 ± 1.0
Stunting (n, %)	70 (30.7)
Underweight* (n, %)	27 (17.2)
**Sex (n, %)**	
Boy	143 (62.7)
Girl	85 (37.3)
**Occupation of household head (n = 227; n, %)**	
Farmer	205 (91.1)
Other^4^	20 (8.9)
**Maternal occupation (n = 227; n, %)**	
Farmer	156 (69.3)
Trader	39 (17.3)
Other^5^	30 (13.3)
**Level of education of household head (n, %)**	
Non-literate	189 (82.9)
Literate	39 (17.1)
**Level of education of mother (n, %)**	
Non-literate	220 (96.5)
Literate	8 (3.5)

Variables are means and standard deviations for continuous variables and frequency and percentages for categorical variables.

^1^BAZ, Body-Mass-Index (BMI)-for-age

^2^HAZ, Height-for-age Z-score

^3^WAZ, Weight-for-age Z-score

^4^Other, including trader and civil servant

^5^Other, including housewife, and civil servant

*Underweight was computed for 157 children with age <10 years (n = 71 were aged ≥ 10 years)

SD = Standard deviation.

### Food consumption pattern and seasonality

Except for small fish eaten whole, < 10% each of the children consumed any other ASF food group in either season (Table 3). When, excluding small fish eaten whole, the percentage of children consuming at least one ASF in the dry season was more than twice the percentage of children consuming it in the rainy season (13.2% vs 4.8%, *P* = 0.002). Compared to the dry season, more children consumed vitamin A-rich dark green leafy vegetables (52.6% vs. 23.3%, *P* < .0001) and vitamin A-rich fruits (64.0% vs. 0.9%, *P* < .0001) in the rainy season. Conversely, more children consumed vitamin A-rich deep yellow, orange and red vegetables in the dry season compared to the rainy season (73.7% vs. 36.4%, *P* < .0001). However, the percentage of children consuming at least one vitamin A-rich fruit/vegetable and vitamin C-rich fruit/vegetable were each significantly higher in the rainy season compared to the dry season (90.7% vs. 81.1%, *P* = 0.003 and 100.0% vs. 96.5%, *P* = 0.004 respectively).

**Table 3:** The frequency and percentage of children consuming each food group stratified by season with its association with season.

Food group	Frequency (%) of children with a score for each food group (n = 228)		*P*-value^1^
	Dry season (October 2010)	Rainy season (May 2011)	
Starchy staples	228 (100.0)	228 (100.0)	-
Legumes and nuts	205 (89.9)	224 (98.3)	0.0002*
Dairy products	20 (8.8)	9 (4.0)	0.03*
Organ meat	0 (0.0)	1 (0.4)	1.00
Eggs	1 (0.4)	0 (0.0)	1.00
Small fish eaten whole	226 (99.1)	227 (99.7)	1.00
Other flesh foods	9 (4.0)	2 (0.9)	0.06
vitamin A rich dark green leafy vegetables	53 (23.3)	120 (52.6)	< .0001*
vitamin A-rich deep yellow, orange and red vegetables	168 (73.7)	83 (36.4)	< .0001*
vitamin A- rich fruits (vitamin A ≥ 120RE/100g	2 (0.9)	146 (64.0)	< .0001*
vitamin C rich vegetables (vitamin C ≥ 9 mg/100g	220 (96.5)	228 (100.0)	0.004*
vitamin C-rich fruits (vitamin C ≥ 9 mg/100g),	4 (1.8)	1 (0.4)	0.37
All other fruits and vegetables	213 (93.4)	207 (90.8)	0.30
At least one animal source food^2^	30 (13.2)	11 (4.8)	0.002*
At least one vitamin A-rich fruit/vegetable	185 (81.1)	207 (90.7)	0.003*
At least one vitamin C-rich fruit/vegetable	220 (96.5)	228 (100.0)	0.004*

^1^ Chi-square test of independence was used to determine the association between the percentage of children consuming a food group and seasonality.

^2^Excludes small whole fish but includes dairy products, organ meat, eggs, and all other flesh foods

*P-value statistically significant at 5% level of significance

The consumption of legumes and nuts increased significantly in the rainy season compared to the dry season (98.3% vs. 89.9%, *P* = 0.0002) whilst the consumption of other fruits and vegetables was marginally higher in the dry season compared to the rainy season (93.4% vs. 90.8%, *P* = 0.30). Furthermore, the consumption of vitamin C-rich fruits was poor in both seasons but vitamin C-rich vegetables were consumed by all the children in the rainy season compared to the dry season (100% vs. 96.5%, *P* = 0.004). Finally, all children consumed starchy staples in both seasons.

When categorizing DDS into low and high, more children had a high DDS in the rainy season compared to the dry season (88.2% vs. 72.4%, *P* < .0001) and the percentage of children with low DDS in the dry season was more than twice the percentage in the rainy season (27.6% vs. 11.8%) (Table 4).

**Table 4:** The frequency and percentage of children with low and high DDS stratified by season.

Food group	Frequency (%) of children (n = 228)		*P*-value^1^
	Dry season (October 2010)	Rainy season (May 2011)	
Low DDS (DDS < 6)	63 (27.6)	27 (11.8)	< .0001*
High DDS (DDS ≥6)	165 (72.4)	201 (88.2)	

^1^ Chi-square test of independence was used to determine the association between the percentage of children with low or high DDS and seasonality

*P-value statistically significant at 5% level of significance.

### Association between DDS and seasonality

The mean DDS of the children was significantly higher in the rainy season compared to the dry season in both crude and multivariate models (Table 5). Adjustment for demographic factors (sex and age) made no difference in the observed means between seasons. Compared to the crude mode, the mean DDS for both seasons increased marginally in the full multivariate model. However, the difference in means between seasons decreased marginally in the full multivariate model compared to the crude model, with the mean DDS remaining significantly higher in the rainy season compared to the dry season (6.54 ± 0.09 vs. 6.00 ± 0.09, *P* < .0001). None of the household covariates was significantly associated (*P* >0.05) with the DDS of the children in either seasons (S1 Table).

**Table 5:** The association between dietary diversity score (DDS) and seasonality among the school-age children in Tolon district of Ghana.

Model	Mean (± SE of mean) Dietary Diversity Score (DDS)		DDS mean difference^1^ (± SE)	*P*-value
	DDS in dry season(October 2010)	DDS in rainy season(May 2011)		
**Crude model**				
Season	5.92 ± 0.06	6.47 ± 0.06	0.56 ± 0.08	< .0001*
**Model 1**				
Season	5.92 ± 0.06	6.48 ± 0.06	0.56 ± 0.08	< .0001*
**Model 2**				
Season	6.00 ± 0.09	6.54 ± 0.09	0.54 ± 0.08	< .0001*

Model 1 was adjusted for age and sex; Model 2 was further adjusted for socioeconomic factors of child’s household: educational level of household head, the occupation of household head and maternal occupation.

^1^Mean DDS in the rainy season minus mean DDS in the dry season

*P-value is significant at 5% level of significance.

From Table 6, the mean score for the consumption of vitamin A-rich fruits and/or vegetables was significantly higher in the rainy season compared to the dry season (1.56 ± 0.07 vs. 1.01 ± 0.07, *P* < .0001). On the contrary, the mean score for consumption of ASF was significantly higher in the dry season compared to the rainy season (1.11 ± 0.03 vs. 1.03 ± 0.03, *P* = 0.008). Lastly, the mean score for the consumption of vitamin C-rich fruits and/or vegetables was similar for both the dry and rainy seasons (1.01 ± 0.02 vs. 0.98 ± 0.02, *P* = 0.17).

**Table 6:** The association between the scores for vitamin A-rich fruits and vegetables, vitamin C-rich fruits and vegetables, animal source foods and seasonality among the school-age children in Tolon district of Ghana.

Model	Mean (± SE of mean)		Differences in means (T_2_ –T_1_) (± SE)	*P*-value
	Dry season (October 2010)	Rainy season (May 2011)		
**Vitamin A-rich fruits and vegetables**				
Crude model (Season)	0.98 ± 0.05	1.53 ± 0.05	0.55 ± 0.06	< .0001*
Model 1 (Season)	0.98 ± 0.05	1.52 ± 0.05	0.55 ± 0.06	< .0001*
Model 2 (Season)	1.01 ± 0.07	1.56 ± 0.07	0.55 ± 0.07	< .0001*
**Vitamin C-rich fruits and vegetables**				
Crude model (Season)	0.98 ± 0.01	1.01 ± 0.01	0.02 ± 0.02	0.17
Model 1 (Season)	0.98 ± 0.01	1.01 ± 0.01	0.02 ± 0.02	0.17
Model 2 (Season)	0.98 ± 0.02	1.01 ± 0.02	0.02 ± 0.02	0.17
**Animal source foods**				
Crude model (Season)	1.12 ± 0.02	1.05 ± 0.02	-0.07 ± 0.03	0.01*
Model 1 (Season)	1.12 ± 0.02	1.05± 0.02	-0.07 ± 0.03	0.01*
Model 2 (Season)	1.11 ± 0.03	1.03 ± 0.03	-0.08 ± 0.03	0.008*

SE, standard error; T_1_, dry season; T_2_ = rainy season;

Model 1 was adjusted for age and sex; Model 2 was further adjusted for socioeconomic factors of child’s household: educational level of household head, the occupation of the household head and maternal occupation

*P-value is significant at 5% level of significance.

## Discussion

In this study, we evaluated the effect of seasonality on the DDS of SAC in rural northern Ghana. In fact, the children had significantly more diverse diets in the rainy season than the dry season, with dietary diversity increasing from a mean of 6.00 food groups (out of 13) in the dry season to 6.54 in the rainy season. Indeed, other studies have also shown food consumption can vary from one season to the next [20–23] and this may directly affect the diversity of diet, especially in the rural Africa setting. Seasonal variations in nutrient intake signifying seasonal differences in food consumption have also been documented in Burkina Faso [26,27]. Our present results are similar to those of Savy et al. [18] who found an increase in DDS from 3.4 ± 1.1 to 3.8 ± 1.5 between the beginning and the end of the food shortage season (*P* < 0.0001) in a sample of Burkinabe women.

Generally, the consumption of vitamin C-rich fruits was poor in both seasons with < 2% of the children consuming any vitamin C-rich fruit in either of the seasons. Studies suggest an intake of fruits and snacks outside the home by SAC is more likely to be omitted by the mothers and caregivers during dietary recalls such as used in our survey [40,41]. Although this may partly explain the poor consumption of vitamin C-rich fruits in both seasons, the children assisted their mothers/caregivers recall their intake outside the home. It was, therefore, less likely that under reporting of fruit intake influenced our results.

We found that a significant proportion of the children consumed vitamin A-rich dark green leafy vegetables and vitamin A-rich fruits in the rainy season than the dry season. Remarkably, < 1% of the children consumed vitamin A-rich fruits in the dry season; this difference may partly be attributed to the intake of shea fruits and mangoes, available only in the early parts of the rainy season. The consumption of vitamin C-rich vegetables was high in both seasons; however, all children consumed vitamin C-rich vegetables in the rainy compared to the dry season. On the contrary, the consumption of vitamin A-rich deep yellow, orange and red vegetables was significantly higher in the dry season compared to the rainy season and may partly be attributed to the availability of orange flesh sweet potatoes which is seasonal and was been harvested at the time of the baseline assessment (October).

Overall, the difference in mean DDS between seasons was mainly due to the difference in consumption of fruits and vegetables between the seasons. Out of 6 food groups which differed significantly between the rainy season and dry season, 4 food groups could broadly be classified as fruits and vegetables with 3 of them significantly higher in the rainy season than the dry season. Additionally, of the 6 food groups which differed significantly between the seasons, 3 were vitamin A-rich fruits/vegetables and 2 of these were significantly higher in the rainy season compared to the dry season. Recent studies from Nigeria [42] and Burkina Faso [27] also found that fruit and/or green leafy vegetable consumption was higher in the rainy season compared to the dry season. In the present study, the difference in consumption of fruits and vegetables between seasons may partly be explained by the seasonal availability of fruits and vegetables in Ghana. A study in Ghana confirmed seasonality influences the consumption of vitamin A-rich foods as vegetables and fruits were found to be more abundant in the rainy season than the dry season [24]. In our experience, the prices of fresh fruits and vegetables are generally higher in the dry season compared to the rainy season, implying poor access for deprived households during the dry season; this may partly account for the differences between the seasons.

Although the results showed that a majority of the children consumed at least one vitamin A-rich fruit and/or vegetable in both seasons, the percentage of children consuming at least one vitamin A-rich fruit and/or vegetable was significantly higher in the rainy season compared to the dry season. Moreover, the mean score for the consumption of vitamin A-rich fruits and/or vegetables was significantly higher in the rainy season compared to the dry season (mean score difference = 0.55 ± 0.07, *P* < .0001). In the present study, the mean score difference between seasons for vitamin A-rich fruits/vegetables was similar to the overall DDS mean difference between seasons (0.55 ± 0.07 vs.0.54 ± 0.08); emphasizing that the difference in mean DDS between seasons was mainly due to the difference in consumption of vitamin A-rich fruits and/or vegetables. A recent study in Bangladesh [43] also reported that differences in DDS by season corresponded with differences in consumption of dark green leafy vegetables and vitamin A-rich fruits/vegetables.

The present analysis did not ascertain whether or not the statistically significant difference in the mean DDS between seasons or the percentage of children consuming each food group is of nutritional importance. Nonetheless, Savy et al [18] showed that a difference in mean DDS between seasons (0.55) similar to that found in our study, has an effect on nutritional status of women in rural Burkina Faso. In another study of younger children (aged 12–59 months) in rural Burkina Faso, Arsenault et al. [27] reported that a difference in mean DDS (mean difference = 0.24) between the pre and post-harvest seasons was associated with a higher micronutrient intake in the post-harvest season compared to the pre-harvest season. Furthermore, Nupo et al. [42] found that a 0.38 difference in mean DDS between seasons is associated with a significant difference in energy, protein and fat intake between seasons in a group of rural Nigerian women. Although the difference in mean DDS across seasons in the present study directly illustrates fluctuations in dietary diversity across seasons among the SAC, it is plausible the difference in mean DDS and/or consumption of vitamin A-rich fruits/vegetables between seasons may influence nutrient intake and consequently dietary adequacy between seasons; but, further studies are needed to establish this.

Adjustment for potential confounders such as age, sex and socio-demographic factors (educational status of household head and occupation of mother/household head) did not change the observed difference between the two seasons. Several studies have shown that household food security and DDS improve with maternal and/or paternal education [18,44,45]. Similarly, it has also been shown that a household head or mother with a better paying job improves DDS [18,19,39]. Although we did not model maternal education due to its homogeneity in the present study, none of the modelled household covariates (education of household head, the occupation of mother and occupation of household head) was significantly associated with the DDS of the children; perhaps a result of the homogeneity of the sample.

In conformity with other studies [22,30,46–48], the results showed poor consumption of ASF in both seasons. Besides “small fish eaten whole” which was consumed by almost every child in both seasons, < 10% each of the children consumed any other ASF in either of the seasons. That withstanding, small fish eaten whole is often consumed in minute quantities in soups and stews in Ghana and may thus be insignificant in improving micronutrient adequacy. Ruel [3] and unpublished data from Ghana confirm small fish eaten whole (anchovies) contributes very little to micronutrient adequacy [49]. This low consumption of ASF in both seasons may have serious consequences for the micronutrient adequacy of the children in either of the seasons as ASF are known to better improve micronutrient adequacy especially for Fe and Zn [50–52] than plant source foods which have poor bioavailability for Fe and Zn [53–55]. In fact, ASF are generally among the most expensive foods [56] in Ghana and may be the reason for their poor consumption in our study population. However, the design of the present study limited its ability to evaluate the effect of the poor ASF intake on the micronutrient adequacy of the children. Similar to other studies [23,57], staples were equally important foods for the children in both seasons as every child consumed a staple (cereals and grains as well as starchy roots and tubers) in both seasons. Even though legumes and nuts were a part of the diet of most of the children in both seasons, the consumption of legumes and nuts was significantly higher in the rainy season compared to the dry season. Liere et al [58], reported that consumption of legumes increases when cereal stocks are becoming depleted; accordingly, the higher legume consumption in the rainy season compared to the dry season may signal a reduction in the quantity of available household cereal stocks. Some studies have reported legumes as good sources of plant protein, with fairly good amounts of Fe and Zn [59,60]. Nonetheless, the current study could not determine whether or not the statistically significant difference in consumption of legumes between seasons is of nutritional importance as we did not assess nutrient intake or dietary adequacy in the present study.

Our study highlights the importance of measuring DDS across the year in order to identify seasonal variations in dietary intake. As a result, researchers who undertake dietary surveys in a context similar to ours need to be mindful of the importance of including data from different seasons. Interventions that aim to improve the diet of young children from low-income, rural communities need to recognize the role of seasonality on diet and to incorporate initiatives to prevent seasonal declines. In designing context-specific interventions to improve the diet of young children, policy makers and programme planners also need to incorporate initiatives that prevent undesirable seasonal declines in dietary diversity.

Likewise, in the design of pre and post-test nutrition evaluation programmes, the effect of seasonality needs to be considered; Swindale and Bilinsky [61] recommended repeated data collection should be undertaken at the same time of the year to avoid seasonal differences. Our findings, therefore, corroborate their recommendation as we have shown that DDS changes across the seasons.

Several strengths and limitations should be taken into account when interpreting the findings of the present study. Firstly, the main strength of the current study lies in its longitudinal design which allowed us to make a comparison of DDS from two seasons among the same individuals. Secondly, the quality of our data is high as we pretested our questionnaire and skilled interviewers who were given a 5-day training undertook the data collection.

On the contrary, residual confounding is always possible in observational studies even with extensive statistical adjustments [62]. Hence, though we adjusted for several potential confounders in this study, we cannot rule out the effect of residual confounding in our present study.

Notably, seasonality is known to influence household food security [43,44,63] which is a key determinant of household dietary diversity as food insecure households often consume low-quality monotonous diets which are low in micronutrients [2,64], but the present study did not include any measure of food security. Nonetheless, food security is well associated with household demographic and socioeconomic characteristics including occupation [26,65,44], education [26,65] and household size [26,65]; thus our adjustment for education, occupation and child compound size may have partly accounted for the effect of household food security.

Our use of a single 24hR and the lack of portion sizes to include a minimum intake for scoring is the main limitation of our dietary assessment method. A single 24hR may not reflect habitual dietary intake and may capture episodic foods that are not typically consumed [66]. There is also evidence that qualitative recalls lead to over reporting and consequently higher scores and misclassification of subjects [16,67]. Thus, it is possible the DDS for both seasons could have been lower than we found if a minimum intake was set for the DDS. More so, the qualitative assessment limited the ability of the present study to determine if the observed difference in mean DDS between seasons reflected seasonal differences in dietary adequacy, especially given that vitamin A-rich foods and legumes were consumed by most children in both seasons and ASF consumption in both seasons was generally poor. On the contrary, the qualitative 24hR was less subject to recall bias, less burdensome to respondents, fast, easy and also conforms to the recall time period used in many dietary diversity studies [2,14,16–18]. Although a food frequency questionnaire is ideal for assessing habitual dietary intake, it is generally associated with more recall bias compared to a 24hR [66,68]; thus, a repeated 24hR may be more appropriate in capturing habitual diet.

The end-line assessment was conducted at the end of the feeding trial, it was therefore unlikely that the daily provision of 430kcal in the intervention trial [29] reduced dietary intake in the end-line. However, the children were treated for helminths infestation after the baseline assessment and 3.5 months into the intervention which could have influenced appetite in the end-line compared to the non-intervention situation. Generally, randomization of children into treatment or control groups had no effect on the DDS at baseline and end-line (results not shown). Caution is however needed in extending the results presented here to larger contexts; at best, the present findings can be extrapolated to rural school-aged children in Northern Ghana where culture and dietary patterns are quite similar.

### Conclusion

In summary, the results of the present study indicate seasonality has an effect on the food consumption pattern and dietary diversity of school-age children in rural Northern Ghana. Although the present study did not evaluate seasonal variability in dietary adequacy, DDS is known to be a proxy indicator of micronutrient intake; thus the seasonal differences in DDS may have an influence on the micronutrient intake of the school-age children between seasons.

## Supporting information

S1 Table Association of household factors with dietary diversity (DDS) for school-aged children in Tolon district by season. DDS, dietary diversity; SE, standard error of mean; ^1^P-value for interaction between household factor and season; for each household factor, Means ± SE with similar superscripts within column do not differ significantly at 5% level of significance.
(DOCX)

S1 Data Dataset for dietary diversity and seasonality. (ZIP)
