# Supplementary material for: Seasonality affects dietary diversity of school-age children in northern Ghana
Source: PLoS One. 2017 Aug 14;12(8):e0183206. doi: 10.1371/journal.pone.0183206 (PMC5555613; doi:10.1371/journal.pone.0183206)
Supplement: S1 Table — DDS, dietary diversity; SE, standard error of mean; 1P-value for interaction between household factor and season; for each household factor, Means ± SE with similar superscripts within column do not differ significantly at 5% level of significance. (DOCX) [file pone.0183206.s001.docx]

**S1 Table. Association of household factors with dietary diversity (DDS) for school-aged children in Tolon district by season**

| **Household factor** | **DDS in dry season (October 2010)** | **DDS in rainy season (May 2011)** | **P^1^ for interaction with season** |
| --- | --- | --- | --- |
|  | **Mean ± SE** | **Mean ± SE** |  |
| **Occupation of household head** |  |  | 0.57 |
| Famer | 5.89 ± 0.06^a^ | 6.45 ± 0.06^a^ |  |
| Other | 6.25 ± 0.16^a^ | 6.65 ± 0. 18^a^ |  |
| **Maternal occupation** |  |  | 0.75 |
| Farmer | 5.95 ± 0.07^a^ | 6.47 ± 0.07^a^ |  |
| Trader | 6.00 ± 0.10^a^ | 6.56 ± 0.13^a^ |  |
| Other | 5.67 ± 0.17^a^ | 6.37 ± 0.16^a^ |  |
| **Educational status of household head** |  |  | 0.63 |
| Literate | 5.79 ± 0.14^a^ | 6.44 ± 0.13^a^ |  |
| Non-literate | 5.94 ± 0.06^a^ | 6.48 ± 0.08^a^ |  |
| **Maternal education** |  |  | 0.87 |
| Literate | 5.88 ± 0.35^a^ | 6.50 ± 0.38^a^ |  |
| Non-literate | 5.92 ± 0.06^a^ | 6.47 ± 0.06^a^ |  |

DDS, dietary diversity; SE, standard error of mean; ^1^P-value for interaction between household factor and season; for each household factor, Means ± SE with similar superscripts within column do not differ significantly at 5% level of significance
