# Supplementary material for: Seasonality affects dietary diversity of school-age children in northern Ghana
Source: PLoS One. 2017 Aug 14;12(8):e0183206. doi: 10.1371/journal.pone.0183206 (PMC5555613; doi:10.1371/journal.pone.0183206)
Supplement: S1 Data — (ZIP) [file pone.0183206.s002.zip › New folder/Labels_and_Coding_for_DDS_and_Seasonality_Dataset.docx]

**Code book for data on DDS and Seasonality in Ghana**

**data** d.DDS_seasonality_Ghana1; set d.DDS_seasonality_Ghana1;

label time="season";

label DDS="Dietary diversity score-DDS";

label hhtotal="child's compound-HH size";

label DDS_cat="DDS median split";

label occu_mum="Maternal occupation recategorised";

label occu_HH="Paternal occupation recategorized";

label edu_HH="Educational status of household head";

label edu_mother="Maternal educational status";

label anyVitA_plant="DDS for any vitamin A-rich plant food";

label anyVitC_plant="DDS for any vitamin C-rich plant food";

label dds_animalfoods=" DDS for animal foods";

label anyVitC="at least 1 of any vitamin C rich plant foods";

label anyVitA="at least 1 of any vitamin A-rich plant foods";

**run;**

**proc** **format**;

value occu_HH

**0**="famer"

**1**="Other including trader, civil servant and other";

value occu_mum

**0**="Others including housewife & civil servant"

**1**="Farmer"

**2**="Trader";

value edu_hh

**1**="non-lliterate"

**2**="Literate";

value edu_mother

**1**="non-literate"

**2**="Literate";

Value DDS_cat

**1**="Low DDS (<6)"

**2**="High DDS (>=6)";

Value Sex

**1**="Boy"

**2**="Girl";

Value Time

**1**="dry season"

**2**="Rainy season";

Value stunting

**1**="Stunted"

**0**="not stunted";

value underweight

**1**="Underweight"

**0**="not underweight"

**.**="undefined(age >10)";

value anyvitA

**1**="Consumption of at least 1 vitamin A-rich plant food"

**0**="no consumption of at least 1 vitamin A-rich plant food";

value anyVitC

**1**="Consumption of at least 1 vitamin C-rich plant food"

**0**="no consumption of at least 1 vitamin C-rich plant food";

value animalfoodcat

**1**="Consumption of at least 1 animal source food"

**0**="no consumption of at least 1 animal source food";

value treatment

**1**="Treatment group"

**2**=" Control group";

**run**;

| **Food group variable label** | **Food group name (Note: score of 1 for consumption of at least 1 food item from group else 0)** |
| --- | --- |
| FoodGroup1 | All starchy stapes |
| FoodGroup2 | All legumes and nuts |
| FoodGroup3 | All diary |
| FoodGroup4 | organ meat |
| FoodGroup5 | eggs |
| FoodGroup6 | small fish eaten whole |
| FoodGroup7 | All other flesh foods and miscellaneous small animal protein |
| FoodGroup8 | Vitamin A rich dark green leafy vegetables |
| FoodGroup9 | Vitamin A-rich deep yellow, orange and red vegetables |
| FoodGroup10 | Vitamin A- rich fruits VA rich ≧120RE/100g as eaten |
| FoodGroup11 | VC rich vegetables VC rich≧9 mg/100g as eaten |
| FoodGroup12 | Vitamin C-rich fruits VC rich≧9 mg/100g as eaten |
| FoodGroup13 | All other fruits and vegetables |
